# Transdiagnostic Individualized Clinically Based Risk Calculator for the Detection of Individuals at Risk and the Prediction of Psychosis: Model Refinement Including Nonlinear Effects of Age

**DOI:** 10.3389/fpsyt.2019.00313

**Published:** 2019-05-09

**Authors:** Paolo Fusar-Poli, Cathy Davies, Grazia Rutigliano, Daniel Stahl, Ilaria Bonoldi, Philip McGuire

**Affiliations:** ^1^Early Psychosis: Interventions and Clinical-detection (EPIC) Lab, Department of Psychosis Studies, Institute of Psychiatry, Psychology and Neuroscience, King’s College London, London, United Kingdom; ^2^OASIS Service, South London and Maudsley NHS Foundation Trust, London, United Kingdom; ^3^Department of Brain and Behavioral Sciences, University of Pavia, Pavia, Italy; ^4^Department of Clinical and Experimental Medicine, University of Pisa, Pisa, Italy; ^5^Department of Biostatistics, Institute of Psychiatry, Psychology and Neuroscience, King’s College London, London, United Kingdom; ^6^Department of Psychosis Studies, Institute of Psychiatry, Psychology and Neuroscience, King’s College London, London, United Kingdom

**Keywords:** psychosis, schizophrenia, at risk, clinical high risk, transdiagnostic

## Abstract

**Background:** The first rate-limiting step for primary indicated prevention of psychosis is the detection of young people who may be at risk. The ability of specialized clinics to detect individuals at risk for psychosis is limited. A clinically based, individualized, transdiagnostic risk calculator has been developed and externally validated to improve the detection of individuals at risk in secondary mental health care. This calculator employs core sociodemographic and clinical predictors, including age, which is defined in linear terms. Recent evidence has suggested a nonlinear impact of age on the probability of psychosis onset.

**Aim:** To define at a meta-analytical level the function linking age and probability of psychosis onset. To incorporate this function in a refined version of the transdiagnostic risk calculator and to test its prognostic performance, compared to the original specification.

**Design:** Secondary analyses on a previously published meta-analysis and clinical register-based cohort study based on 2008–2015 routine secondary mental health care in South London and Maudsley (SLaM) National Health Service (NHS) Foundation Trust.

**Participants:** All patients receiving a first index diagnosis of non-organic/non-psychotic mental disorder within SLaM NHS Trust in the period 2008–2015.

**Main outcome measure:** Prognostic accuracy (Harrell’s *C*).

**Results:** A total of 91,199 patients receiving a first index diagnosis of non-organic and non-psychotic mental disorder within SLaM NHS Trust were included in the derivation (33,820) or external validation (54,716) datasets. The mean follow-up was 1,588 days. The meta-analytical estimates showed that a second-degree fractional polynomial model with power (−2, −1: age1 = age^−2^ and age2 = age^−1^) was the best-fitting model (*P* < 0.001). The refined model that included this function showed an excellent prognostic accuracy in the external validation (Harrell’s *C* = 0.805, 95% CI from 0.790 to 0.819), which was statistically higher than the original model, although of modest magnitude (Harrell’s *C* change = 0.0136, 95% CIs from 0.006 to 0.021, *P* < 0.001).

**Conclusions:** The use of a refined version of the clinically based, individualized, transdiagnostic risk calculator, which allows for nonlinearity in the association between age and risk of psychosis onset, may offer a modestly improved prognostic performance. This calculator may be particularly useful in young individuals at risk of developing psychosis who access secondary mental health care.

## Introduction

**Figure 1:**
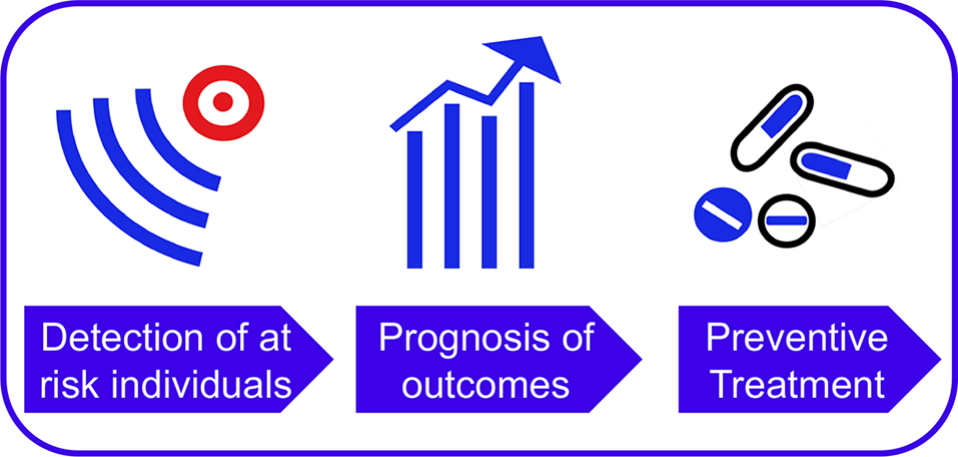
Core clinical and research components for an effective prevention of psychosis. Figure reproduced with permission (CCBY 4.0) from Ref. (2).

Primary indicated prevention in individuals meeting a Clinical High Risk state for Psychosis [CHR-P (1)] entails three stepped core components: efficient detection of individuals at risk, an accurate prognosis of outcomes, and an effective preventive treatment that can impact the course of the disorder (**Figure 1**) (3).

The first rate-limiting step is the detection of children, adolescents, and young adults aged 8–40 (4) (more frequently 14–35) (5) who may be at risk of developing psychosis. Their detection is based on recruitment campaigns (6) that filter individuals who have accumulated several risk factors (7) for the development of psychosis, thus enriching the level of risk. The actual ability of specialized CHR-P clinics (5) to detect individuals at risk for psychosis is limited. The first study to explore this issue has estimated that only about 5% of individuals who will later develop a first episode of psychosis in secondary mental health care was detected by the local CHR-P specialized clinics at the time of their CHR-P stage (8, 9). Even frontline youth mental health services can detect only about 12% of first-episode cases (10). It is therefore clear that we need to improve our ability to detect CHR-P individuals in secondary mental health, primary care, and the community. To overcome this substantial challenge, a clinically based, individualized, transdiagnostic risk calculator has been developed and externally validated twice (2, 8, 11). This calculator leverages electronic health records to screen secondary mental health care trusts at scale. Secondary mental health care is characterized by an incidence of psychosis of 3% at 6 years, which is higher than the risk of psychosis of 0.62 at 6 years in the local general population [see eFigure 1 published in Ref. (8)]. The calculator uses as key predictors ICD-10 index diagnosis [because it allows several diagnostic spectra, it is termed as transdiagnostic; see Ref. (12)], age, gender, age by gender, and ethnicity, which have been selected on the basis of *a priori* clinical knowledge (13, 14).

Specifically, age has been included because it is one of the most established sociodemographic risk factor for psychosis (15). In the original version of the transdiagnostic risk calculator, the impact of age on the risk of psychosis onset has been defined in linear terms. However, converging evidence from independent meta-analyses (15, 16) suggests that age may exert a nonlinear effect on the risk to psychosis onset. For example, a recent umbrella review (17) found that the risk for psychosis increases from childhood to young adulthood, peaks between 15–35 years (16), and then declines after this age, independent of gender (age older than 35 was found to be a protective factor) (16). It is thus possible that the use of nonlinear definitions of age would better represent the impact of this factor on the onset of psychosis, in particular for the youngest CHR-P groups (18).

In this study, we test such a hypothesis. We first employ independent meta-analytical data to investigate the epidemiological association with age and risk of developing psychosis onset in the general population. We then use this information to refine the original transdiagnostic risk calculator and to test whether its prognostic accuracy would improve. The results of this study can inform future risk prediction research in the field of early psychosis.

## Methods

### Data Source

South London and the Maudsley (SLaM) is a National Health Service (NHS) Mental Health Trust. SLaM provides secondary mental health care to a population of approximately 1.3 million residents of four London boroughs (Lambeth, Southwark, Lewisham, and Croydon). The Trust is effectively digitalized and paper-free (19), and all patients have a personal electronic clinical record. It is a legal requirement for SLaM health care professionals to keep these records up to date (19). The SLaM register contains the full clinical records of all patients, which are continually updated throughout their care, regardless of discharges from or referrals to other services. A Clinical Record Interactive Search (CRIS) tool (19) was implemented to facilitate searching and retrieval of full but anonymized clinical information for research purposes (19). Because the CRIS tool draws directly from these electronic health records, it provides valuable “real-world” and “real-time” information on routine mental health care (20). CRIS has already been used in At Risk Mental State (ARMS) studies (21) as well as in over 70 previous publications (22–24). CRIS-related methods and descriptive data of the SLaM cohort have been extensively detailed (19, 20, 25–28).

### Study Population

As indicated in the original study and in its replication (11), all individuals accessing SLaM services in the period 1st January 2008 to 31st December 2015, and who received a first index primary diagnosis of any non-organic and non-psychotic mental disorder, were initially considered eligible. We then excluded those who developed psychosis in the 3 months immediately following the first index diagnosis. Approval for the study was granted by the Oxfordshire Research Ethics Committee C. Because the dataset was made up of de-identified data, informed consent was not required (19).

### Variable Definitions

The outcome (risk of developing any psychotic disorder), predictors, and time to event were automatically extracted using CRIS (19). Predictors (index diagnosis, age, gender, ethnicity, and age by gender interaction) were preselected on the basis of previous meta-analytical clinical knowledge, as recommended (29) [see the original study for full details (8)]. Age was entered as a continuous predictor and measured at the time of the index diagnosis, while self-assigned ethnicity and index diagnoses were operationalized as previously indicated (2). The outcome (risk of developing any psychotic disorder) was defined as the emergence of the first ICD-10 (30) primary diagnosis of non-organic psychotic disorder, occurring at least 3 months after the index diagnosis as recorded in the local electronic medical records: schizophrenia spectrum psychoses [schizophrenia (F20.x, except F20.4/F20.5), schizoaffective disorder (F25.x), delusional disorders (F22.x, F24), acute and transient psychotic disorders (F23.x)], unspecified nonorganic psychosis (F28/F29), psychotic disorders due to psychoactive substance use [(F10–F19).5], and affective psychoses [mania with psychotic symptoms (F30.2), bipolar affective disorder with psychotic symptoms (F31.2, F31.5), and depression with psychotic symptoms (F32.3/F33.3)]. Accordingly, baseline ICD-10 psychotic disorders were excluded, with the exception of acute and transient psychotic disorders (F23.x), which are, by definition, clinically remitting and non-psychotic within 3 months (short-lived). The rationale for including the ATPD is due to the fact that this group is prognostically similar to the Brief Limited Intermittent Psychotic Symptom (BLIPS) or Brief Limited Psychotic Symptoms (BIPS) subgroups of the CHR-P construct [for details on these competing operationalization, see previous publications on the diagnostic and prognostic significance of BLIPS (31, 32)]. On a diagnostic level, about two-thirds (68%) of BLIPS meet ATPD criteria (31). Individuals with ATPDs/BLIPS are also those more likely to present unmet clinical needs because they are too ill for CHR-P services and not enough ill for first-episode services (33, 34).

The follow-up (time to event) began 3 months after the date of the index diagnosis within SLaM, censored at 1st April 2016. This lag period was chosen to allow patients sufficient time after their index diagnosis to meet the ICD-10 duration criterion for ATPD.

### Statistical Analysis

This original clinical register-based cohort study was conducted according to the REporting of studies Conducted using Observational Routinely-collected health Data (RECORD) Statement (35).

#### Development and Validation Databases

**Figure 2:**
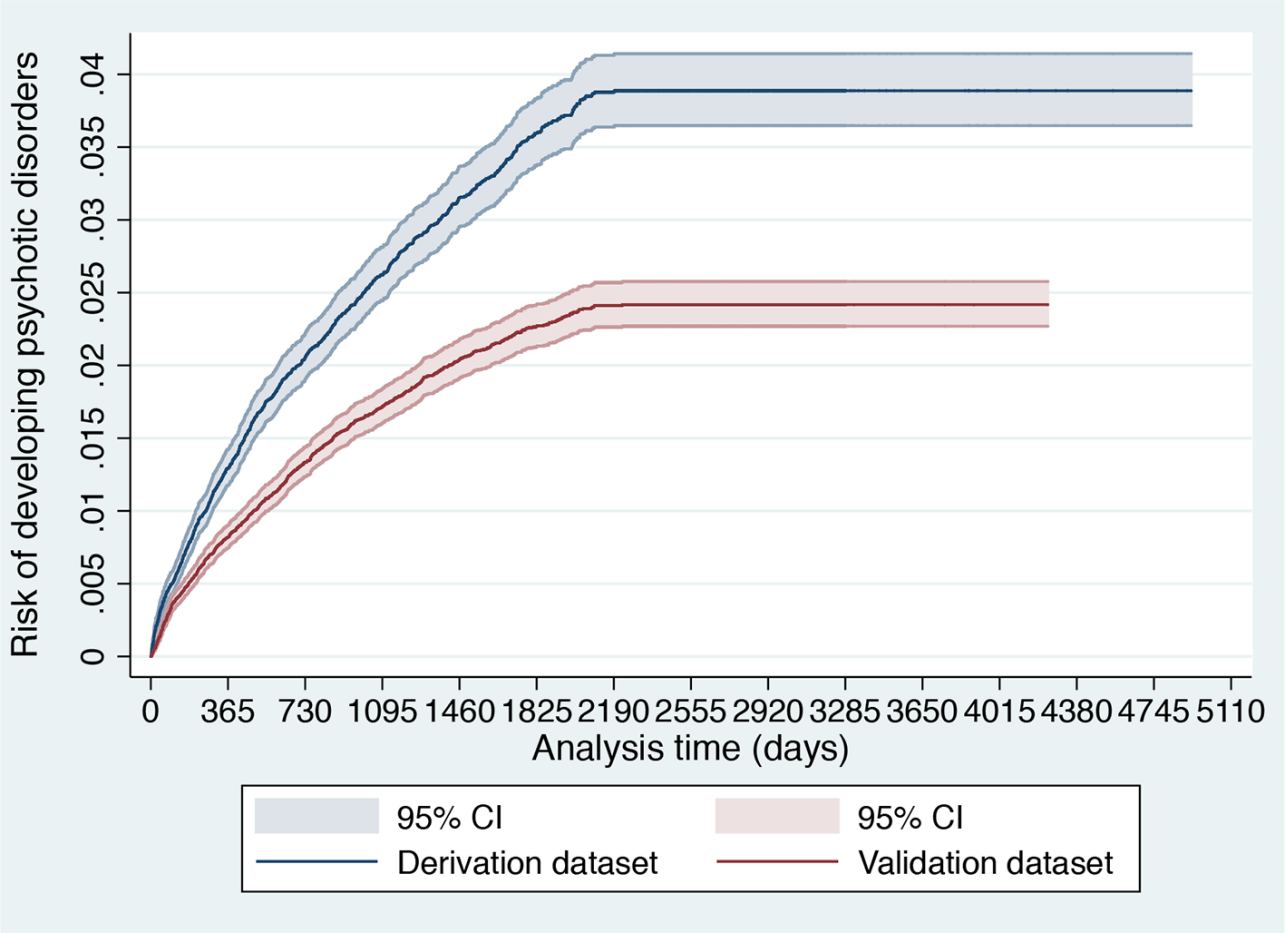
Cumulative incidence (Kaplan–Meier failure function) for risk of development of psychotic disorders with 95% CIs in 91,199 patients accessing SLaM during 2008–2015 stratified across the derivation and validation datasets.

**Figure 3:**
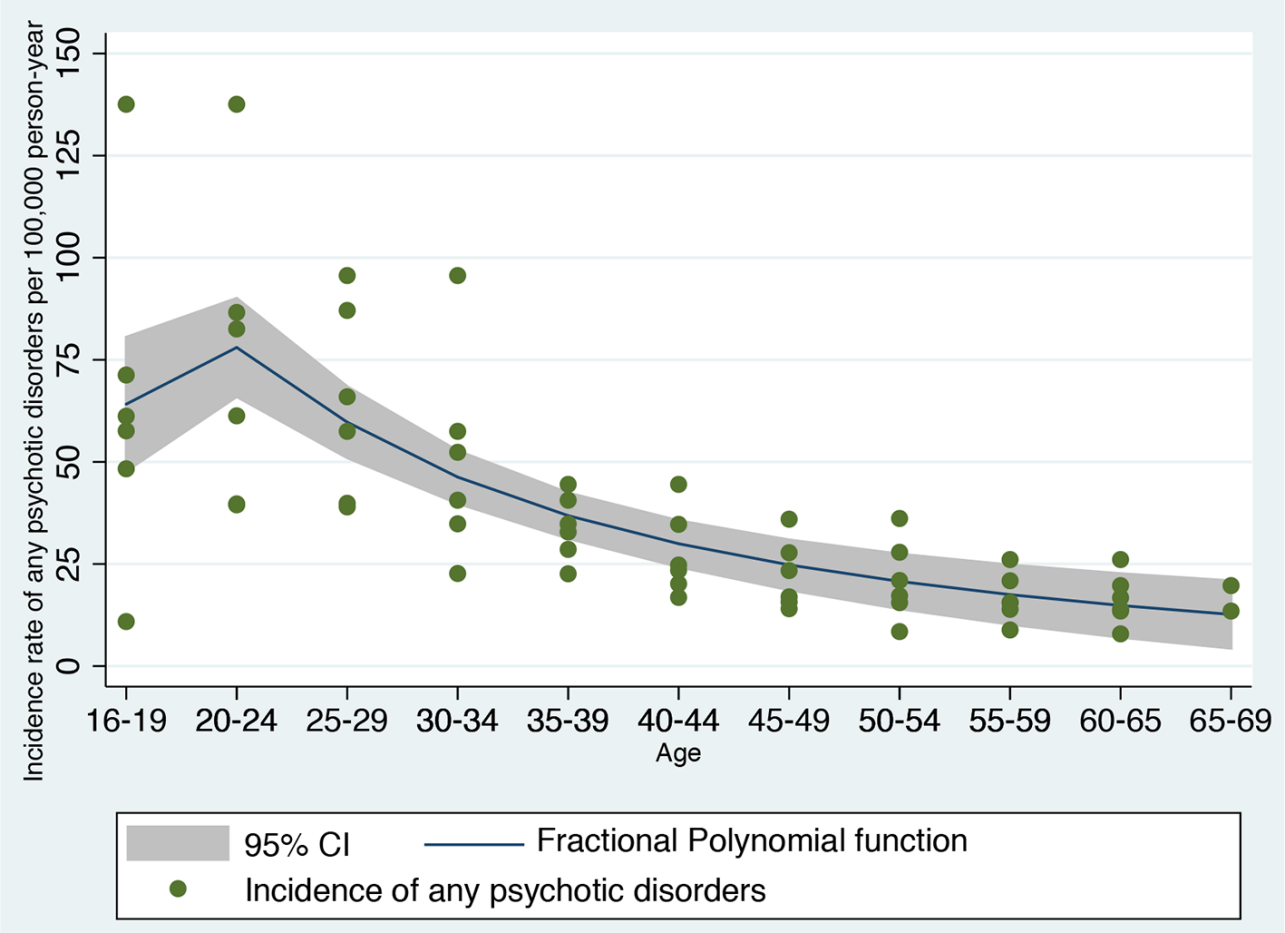
Fractional polynomial analysis investigating the nonlinear association between incidence rate of developing any psychotic disorder in England and age bands, computed on meta-analytical data previously published (42).

The same development and validation databases were used in the current study. Because of significant sociodemographic differences between the SLaM boroughs [from Ref. (20): see **Table 1** and **Figures 2** and **3**], we used a nonrandom split-sample approach using the geographical location to define the development and external validation (36), with the Lambeth and Southwark cases in the derivation sample and all other cases in the validation sample. The use of nonrandom split based on geographical location was based on the substantial sociodemographic differences across these urban areas (19), which can optimize the estimation of external prognostic accuracy (13). Model development and validation followed the guidelines of Royston and Altman (37), Steyerberg et al. (38), and the Transparent Reporting of a multivariable prediction model for Individual Prognosis Or Diagnosis (TRIPOD) (36).

**Table 1:** Sociodemographic characteristics of study population, including the derivation and validation dataset, from Ref. (8).

Age (years)^(a)^	Study population (n = 91,199)^(a)^		Derivation dataset (n = 33,820)		Validation dataset (n = 54,716)		Validation vs. derivation	
	Mean	SD	Mean	SD	Mean	SD	*t*	*P*
	32.97	18.63	34.4	18.92	31.98	18.54	18.73	<0.001
	Count	%	Count	%	Count	%	X^2^	*P*
Gender							13.37	<0.001
Male Female Missing	46,404	50.88	17,303	48.81	27,302	49.90		
	44,761	49.08	16,507	51.16	27,398	50.07		
	34	0.04	10	0.03	16	0.03		
Ethnicity							50.21	<0.001
Black White Asian Mixed Other Missing	14,327	15.71	6,879	20.34	7,023	12.84		
	55,679	61.05	18,627	55.08	35,392	64.68		
	3,830	4.20	1,129	3.34	2,608	4.77		
	3,319	3.64	1,306	3.86	1,957	3.58		
	5,700	6.25	3,466	10.25	2,084	3.81		
	8,344	9.15	2,413	7.13	5,652	10.33		
Index diagnosis							48.20	<0.001
CHR-P	368	0.40	314	0.93	50	0.09		
Acute and transient psychotic disorders	1,370	1.50	553	1.64	725	1.33		
Substance use disorders	14,689	16.11	7,149	21.14	6,507	11.89		
Bipolar mood disorders	2,558	2.80	950	2.81	1,526	2.79		
Non-bipolar mood disorders	15,496	16.99	6,302	18.63	8,841	16.16		
Anxiety disorders	24,770	27.16	8,235	24.35	15,960	29.17		
Personality disorders	3,562	3.91	1,286	3.80	2,116	3.87		
Developmental disorders	5,192	5.69	1,412	4.18	3,706	6.77		
Childhood/adolescence onset disorders	13,984	15.33	4,200	12.42	9,629	17.60		
Physiological syndromes	7,053	7.73	2,555	7.55	4,424	8.09		
Mental retardation	2,157	2.37	864	2.55	1,232	2.25		

(a) SLaM boroughs used to define the derivation (Lambeth and Southwark) and validation (any other) datasets: Lambeth and Southwark 33,820 (37.08%), any others 54,716 (60.00%), missing 2,663 (2.92%).

CHR-P, Clinical High Risk state for Psychosis.

Several steps were followed to explore similarities and differences between the derivation and validation dataset. First, sociodemographic characteristics were compared. Second, the cumulative incidence of psychosis across the two databases was estimated with the Kaplan–Meier failure function (1 − survival) and Greenwood 95% CIs (39). Then, we visually compared the two Kaplan–Meier failure functions. If the visual inspection of the curves reveals that they vary noticeably or if there are differences in their shapes, systematic differences within the study populations may be indicated (40). Third, we also reported the spread (SD) and the mean of the prognostic index of the original model in the two databases. An increased (or decreased) variability of the prognostic index would indicate more (or less) heterogeneity of case-mix between the two databases and, therefore, of their overarching target populations (41). Differences in the mean prognostic index indicate differences in overall (predicted) outcome frequency, reflecting case-mix severity between the two databases (and revealing the model’s calibration-in-the-large in the validation database) (41).

#### Development of the Revised Model

In line with the rationale detailed in the Introduction, we tested here a refined version of the original model. While age was entered as a continuous predictor in the original model, we employed here a revised model that additionally allowed for nonlinearity in the association between age and risk of psychosis. All of the other predictions remained unchanged.

First, the type of nonlinear function linking age and risk of psychosis was defined *a priori* on the basis of independent data not based on the current derivation dataset. We used a robust systematic review and meta-analysis reporting on the incidence of any psychotic disorders in England (15, 42). Using *a priori* knowledge to build a prognostic model is a robust and recommended approach, which can minimize the risk of overfitting (13). Overfitting would be high if we would have used the same dataset to estimate the type of nonlinear function linking age and risk of psychosis, and then fitted a prognostic model on the same data (13). Therefore, we extracted Figure 4.4 from the previously published meta-analysis, which was reporting the meta-analytical incidence of all clinically relevant psychoses by age and gender, reported in Ref. (42). This figure represents the most updated and largest epidemiological estimate of the incidence of psychotic disorders in England. The figure was then digitalized into a.png picture file so that each curve angle could be identified by its pixel coordinate. A free image manipulation software DigitizeIt (http://www.digitizeit.de/) was then used to identify the pixel coordinates for each curve. The meta-analytical data thus extracted were imported into an electronic dataset and age ranges were remapped to a linear (ordinal) variable. We then applied a fractional polynomial (FP) approach to identify the best nonlinear model fitting the observed outcomes. Although there are alternative modeling strategies to deal with nonlinear effects such as quadratic regression and spline-based models, FP is probably the most powerful statistical method to capture the nonlinear effect of variables (43, 44). FP of degree *m* for the variable *X* with powers *p*1…*pm* is given by FP*m*(*X*) = β_1_ *X^p^* ^1^+….β_m_ *X^pm^* (for simplicity, we suppress the constant term, *β*_0_). Usually, *m* = 1 or *m* = 2 is sufficient for a good fit. Therefore, there are two classes of FP: first-degree fractional polynomials (FP1) and second-degree fractional polynomial (FP2) (45). Powers *p*1 and *p*2 are taken from a special set *S* = {–2, –1, –0.5, 0, 0.5, 1, 2, 3} (43). Thus, the first-degree polynomial model (FP1) is β_1_ *X^p^* ^1^ and performs 8 tests and detects whether the fit is improved by a power transformation of the variable *X* in *Xp*. FP with a value of *p* = 1 indicates a linear regression while *p* = 0 indicates that a logarithmic transformation is required for optimum linear modeling of the variable *X*. The second-degree polynomial models (FP2) are an extension to *β*_1_ *_X_p* ^1^ + *β*_2_ *_X_* *p* ^2^, which compares 36 different power combinations. The case of (*p*_1_ = 1, *p*_2_ = 2) is equivalent to quadratic regression. The case *p*_1_ = *p*_2_ is known as repeated power model and has been defined as β_1_ *_X_p*+ β_2_ *_X_p*Ln *X* (44).

The STATA package “fp” was then used to isolate the FP powers that were best fitting the meta-analytical data and thus representing the epidemiological impact of age on the risk of psychosis.

The package fp performs FP comparisons across the powers (−2 −1 −0.5 0.5 1 2 3) and two degrees. Therefore, the linear predictor is included in the comparisons. Different FP models are compared and the corresponding model deviance, defined as twice the negative log-likelihood, is estimated. Under linear regression, a partial *F* test comparing the model is performed and a X^2^ statistic is computed and we selected the best-fitting model as the model with the lowest deviance. Overall, this approach delivered an epidemiological estimate of the relationship between age and psychosis, at a meta-analytical level. In a subsequent step, we tested the real-world benefit of the specific revised model. To further illustrate the type of FP that was selected through this method, we plotted the FP function that was fitted to the epidemiological meta-analytical data and reported its regression coefficients. However, as indicated above, our aim was to use *a priori* knowledge to identify the type of FP function and not specifically the regression coefficients of the FP.

We then used Cox proportional hazards multivariable complete-case analyses to evaluate the effects of the revised model on the development of non-organic ICD-10 psychotic disorders and time to development of psychosis, after checking the proportional hazards assumption (46). This model with all preselected predictors was first fitted to the derivation data to estimate the optimal regression coefficients. Performance diagnostics of individual predictor variables in the derivation dataset were explored with Harrell’s *C* index (37), which is similar to the area under the receiver operating characteristic curve. Values of 0.9–1.0 are considered outstanding, 0.8–0.9 excellent, and 0.7–0.8 acceptable (47). We then generated individual prognostic scores, allowing a prognostic index for risk of psychosis onset to be developed in the derivation dataset (48).

#### External Validation of the Revised Model

The regression coefficients as estimated in the derivation dataset were then applied to each case in the external validation dataset, to generate the PI in the validation dataset. Overall model performance [the distance between the predicted outcome and actual outcome (38)] was assessed with the Brier score [the average mean squared difference between predicted probabilities and actual outcomes, which also captures calibration and discrimination aspects (38)]. A lower score indicates higher precision and less bias, but interpretation depends on the incidence of the outcome (38). Overall performance was further investigated with Royston’s modification of Nagelkerke’s *R* ^2^ (indexing the proportion of variation explained by the model through the str2d STATA package) (49). Calibration [the agreement between observed outcomes and predictions (38)] was assessed with the regression slope of PI (38) (which also captures discrimination and model fit) (37) and with the calibration-in-the-large (38).

Discrimination [accurate predictions discriminate between those with and those without the outcome (38)] was addressed with Harrell’s *C* index (primary prognostic accuracy outcome) (37) and with the discrimination slope [difference in mean of predictions between outcomes (38)].

To test whether the refined model was associated with improved performance compared to the original model, Harrell’s *C* index was compared across the two models in the validation dataset, using the “lincom” function as detailed in an established procedure (50). Recent studies indicate that unbiased and precise estimation of performance measures can be achieved with a minimum of 100 events in the external validation dataset (51).

All analyses were conducted in STATA 14.

## Results

### Sociodemographic and Clinical Characteristics of the Derivation and Validation Datasets

As indicated in the original study (8), of 92,227 patients receiving a first index diagnosis of non-organic and non-psychotic mental disorder within SLaM in the period 2008–2015, 91,199 fulfilled the study inclusion criteria and were included in the derivation or validation datasets. The mean follow-up was 1,588 days (95% CI, 1,582–1,595) with no differences between the derivation and validation datasets. The core characteristics of the sample are presented in **Table 1**. The cumulative incidence across the derivation and validation datasets is represented in **Figure 2** and indicates a lower risk of psychosis in the validation dataset compared to the derivation dataset. The mean values of the prognostic index were −1.32 (SD, 0.896) in the derivation dataset and −1.581 (SD, 0.888) in the validation dataset, indicating a slightly reduced variation in the validation dataset and some case-mix.

### Model Development

#### Fractional Polynomial Analysis

The FP analysis in the independent meta-analytical data confirmed that a second-degree FP (FP2) model with power (−2, −1) was the best-fitting model minimizing the deviance (deviance = 545.188, SD = 20.14, F = 33.55, *P* < 0.001, **Figure 3**). A second-degree (FP2) model with power (−2, −1) was fitting better the meta-analytical data compared to the linear model (FP1: age1 = age^−1^, deviance = 552.066, SD = 21.10), which was used as the original prognostic model (8), to a first degree (FP1) with power 0.5 (age1 = age^0.5^, deviance = 551.33, SD = 20.982) and to the omission of this predictor (deviance = 592.276, SD = 29.951). Accordingly, we generated two (unscaled) variables: age1 = age^−2^ and age2 = age^−1^. The resulting nonlinear meta-analytical polynomial function is depicted in **Figure 3**. The fitted function in **Figure 3** was incidence of any psychotic disorders per 100,000 person-year = −11.27 − 206.35*age^−2^ + 281.75*age^−1^.

#### Model Performance in the Derivation Dataset

In the derivation dataset, there were 1,001 transitions to psychosis. The multivariable model significantly predicted psychosis onset (likelihood ratio chi-square test = 1878, *P* < 0.001, **Table 2**).

**Table 2:** Statistics for individual predictor variables in the refined multivariable Cox proportional hazards regression analysis of risk for psychosis in the derivation dataset and individual predictor variables for the original model.

Predictor	Refined model				Original model
	*β* Coefficient	95% CI		*P*	*β* Coefficient
Age 1 (years)^(a)^	−469.25780	–	–	0.010	–
Age 2 (years)^(b)^	17.97139	−23.87948	59.82225	0.400	–
Gender					
Male	0.83976	0.49192	1.18760	<0.00	0.56818
Female	1				1
Age by gender (male)	−0.01887	−0.02756	−0.01018	<0.00	−0.01219
Age (years)	0.00186	−0.01290	0.01663	0.805	0.01171
Ethnicity					
White	1				1
Black	1.05500	0.90909	1.20091	<0.00	–1.03792
Asian	0.47955	0.16086	0.79824	0.003	0.51434
Mixed	0.66630	0.30616	1.02643	<0.00	0.60440
Other	0.34255	0.12499	0.56010	0.002	0.40810
Index diagnosis					

(a) age1 = age^−2^.

(b) age2 = age^−1^.

Beyond the age effect, there were no substantial changes in the significance of the predictors compared to the original model: male gender (relative to females), Black, Asian, mixed, and other ethnicities (relative to White ethnicity) remained significantly associated with an increased risk of psychosis (**Table 2**). Across males, the risk of psychosis remained negatively associated with increasing age (**Table 2**). Since the age by gender interaction was included in the model, age was also retained as a linear predictor, which was not more significant. Compared with the reference CHR-P designation, all of the other ICD-10 mental disorders were still associated with a lower risk of developing psychosis (**Table 2**). The exceptions were bipolar mood disorders and acute and transient psychotic disorders that showed a comparable and higher risk of psychosis than the CHR-P, respectively (**Table 2**). Model diagnostics using the *C* index are detailed in **Table 2**. The model showed excellent overall apparent performance (excellent discrimination, *C* index = 0.814) and explained approximately 77% of the observed variation (**Table 3**).

**Table 3:** Performance of the refined risk calculator—including the nonlinear effect of age—for transdiagnostic prediction of psychosis in secondary mental health care.

Performance measure	Derivation			Validation		
	**95% CI**			**95% CI**		
*Overall*						
Brier^(a)^	0.027			0.018		
*R* ^2^ (mean, 95% CI)	0.771	0.734	0.806	0.743	0.701	0.781
*Discrimination*						
Harrell’s *C* (mean, 95% CI)	0.814	0.800	0.829	0.805	0.790	0.819
Harrell’s *C* difference with the original model^(a)^	0.014	0.080	0.020	0.0136	0.060	0.021
Discrimination slope^(b)^	0.174	0.177	0.168	0.121	0.125	0.118
*Calibration*						
Calibration-in-the-large	0			0.03		
Calibration slope (mean, 95% CI)	1			0.971	0.932	1.011

(a) Harrell’s C in the original model: derivation, 0.80; validation, 0.79^7^.

(b) at 10 years.

Compared to the original model, the refined model was associated with a modest (Harrell’s *C* change = 0.014) but significant (95% CI from 0.008 to 0.020, *t* = 4.63, *P* < 0.001, **Table 3**) improvement in performance.

#### Model Performance in the Validation Dataset

In the validation dataset, the refined model was associated with a relatively lower Harrell’s *C* (and explained 74% of the observed variation), which, however, remained excellent: 0.805 (95% CI from 0.790 to 0.819). This was likely due to the lower risk of psychosis and reduced variation in the validation database. However, the refined model was still characterized by a modest (Harrell’s *C* change = 0.0136) and significant (95% CIs from 0.006 to 0.021, *t* = 3.56, *P* < 0.001) improvement in performance, compared to the original model.

## Discussion

This study advances knowledge in the field of the detection of individuals at risk for psychosis using automated methods that employ electronic health records. Meta-analytical FP analyses demonstrated that age has a nonlinear effect on the risk of psychosis onset. This evidence was used to refine a previously validated individualized clinically based, transdiagnostic risk calculator. The refined model demonstrated modest but significantly superior prognostic accuracy than the original model in the external validation.

The core aim of this study was to refine an automated detection tool to identify individuals at risk of developing psychosis at scale. Overall, the improved prognostic accuracy was modest in magnitude, although statistically significant. Because of the limited size of the improvement, it is unlikely that it will be associated with substantially higher clinical benefits. Yet, medical knowledge proceeds by incremental steps that can eventually deliver substantial advancements. In this light, methodological guidelines recommend updating and refining existing prognostic models through several iterations, rather than dropping the model and developing new ones from scratch (13). In fact, the current refined version of this prognostic model may show higher prognostic stability in other clinical scenarios, for example, in young CHR-P populations aged 16–20—as indicated in **Figure 3**, the polynomial function may show a better fitting than the linear function in this specific age period. Improving the prognostic accuracy for the prediction of clinical outcomes in the young CHR-P population is particularly important because current CHR-P psychometric interviews do not perform well in these patients. Accumulating evidence has demonstrated a dilution of transition risk in underage CHR-P patients compared to older CHR-P samples (18). This effect may cause instability in prognostic models and lack of generalizability. Conversely, the current refined prognostic model may be more flexible and, by capturing nonlinear as well as linear effects of age in the youngest groups, may be more generalizable across different age groups. Notably, the original prognostic model was not only transdiagnostic but also ageless. As such, it has the potential to be applied to individuals at risk of psychosis over the neurodevelopmental period, provided they have received an initial ICD-10 diagnosis while accessing secondary mental health care. Because of this characteristic, the refinement of the current model to incorporate epidemiological effects of age may be associated with some pragmatic utility. Clearly, this would need to be demonstrated in future cohort studies of young CHR-P samples. These results may also have other relevant impacts.

On a conceptual level, improved detection of individuals at risk for psychosis is urgently needed because, as detailed in the Introduction, current detection strategies are highly inefficient. More to the point, it is also essential to standardize the way individuals at risk for psychosis are recruited for undergoing a CHR-P assessment. In fact, individuals meeting CHR-P criteria display functional impairments (52) and a 20% risk of developing psychosis at 2 years (53) [but not an increased risk of developing other non-psychotic mental disorders (54, 55)]. The meta-analytical prognostic accuracy of the CHR-P instruments is excellent [area under the curve (AUC) at 3 years: 0.9] and is comparable to that of other preventive paradigms in organic medicine (56). Yet, such an excellent prognostic accuracy is mostly due to CHR-P instruments’ ability to rule out a state of risk for psychosis. In fact, testing negative at a CHR-P assessment leads to a 10-fold decrease in the [posttest (57)] risk of developing psychosis (negative likelihood ratio of 0.01) (56, 57). Conversely, the CHR-P instruments’ ability to rule-in a state of risk for psychosis is modest. In fact, testing positive a CHR-P assessment leads only to a 1.8-fold increase in the [posttest (57)] risk of developing psychosis (positive likelihood ratio of 1.8) (56, 57). The consequence is that CHR-P instruments’ prognostic accuracy is excellent provided samples to which they are applied undergo some risk enrichment before the assessment [termed as pretest level of risk (57)]. In fact, CHR-P instruments do not work well when they are applied outside clinical samples that have already undergone some pretest risk enrichment (58, 59). This is traditionally obtained during the recruitment or detection phase, which is mostly unstandardized. For example, when individuals are recruited from mental health services, they accumulate several risk factors for psychosis (7) and their level of risk raises to 15% at 3 years worldwide (6). Such a pretest level of risk for psychosis is substantially higher compared to the 0.43% 3-year risk of the local age-matched general population (14, 60). These considerations explain the most important challenges of the CHR-P paradigm. For example, the lack of statistical power because of the poor level of psychosis risk led to underpowered and negative randomized controlled trials in this population (61). Furthermore, small sample sizes in CHR-P trials are associated with inaccurate estimates and large 95% confidence intervals that have been recently observed in meta-analyses of CHR-P treatments (62). The main problem is that it is currently not possible to control recruitment strategies in a systematic fashion. For example, owing to intense outreach campaigns in the community, the actual posttest risk of psychosis in CHR-P samples has been declining from 29% [2012 (63)] to 20% [2016 (53)] worldwide. There are, however, some exceptions to this phenomenon. For example, in the Outreach and Support in South London CHR-P service (5), transition risk has not been declining over time. This is due to the fact that recruitment strategies have overall maintained a stable pretest risk enrichment (64). These points altogether corroborate the scientific rationale for developing innovative detection and recruitment strategies that could guarantee a clinically meaningful level of pretest risk enrichment in this field.

On an empirical level, this study has some additional impact on the field of prognostic modeling for early psychosis. First, to the best of our knowledge, our approach is the first one to date that has ever attempted to estimate the age effect on the probability of psychosis onset at a meta-analytical level. Because this finding is *per se* robust, future prognostic modeling studies in the field of early psychosis that are considering using age as a predictor could further consider defining it in polynomial terms (age1 = age^−^ *2* and age2 = age^−^ *1*), as proposed here. Importantly, in our model, age was also retained as a linear predictor because the age by gender interaction was included in the model. Second, there are some practical implications relating to the real-world implementation of the individualized transdiagnostic risk calculator. A feasibility implementation study is ongoing in South London and could consider using the refined version of the calculator (2). The refinement of prognostic models and their updating to facilitate their real-world clinical usage is a recommended procedure to improve their prognostic performance as opposed to continuously developing new models that eventually do not enter in clinical routine (13).

Limitations of this study are mostly inherited from the original model and are fully detailed in the previous publications (8, 11). In brief, our diagnoses have high ecological but unclear psychometric validity. As such, it is possible that the model is charting out relationships that reflect diagnostic practice within the United Kingdom. Future external replication studies are needed to establish the generalizability of this model outside the United Kingdom. Randomized clinical trials or economic modeling are needed to assess whether our risk calculator effectively improves patient outcomes.

## Conclusions

The use of a refined version of the clinically based, individualized, transdiagnostic risk calculator, which allows for nonlinearity in the association between age and risk of psychosis onset, may offer a modestly improved prognostic performance. This calculator may support an improved detection of individuals at risk of developing psychosis in secondary mental health care, in particular for the young population.

## Ethics Statement

The study uses anonymized data and has received REC approval.

## Funding

This study was supported by the King’s College London Confidence in Concept award from the Medical Research Council (MRC) (MC_PC_16048) to PFP. These funding bodies had no role in the design of the study, collection, and analyses.

## Author Contributions

PFP conceived the study and conducted the analyses with the supervision of DS. GR prepared the database and contributed to the analyses. CD extracted the meta-analytical data. The other authors contributed to the revision of the manuscript.

## Conflict of Interest Statement

The authors declare that the research was conducted in the absence of any commercial or financial relationships that could be construed as a potential conflict of interest.
